# Using Patient-Based Computational Fluid Dynamics for Abdominal Aortic Aneurysm Assessment

**DOI:** 10.3390/bioengineering12121380

**Published:** 2025-12-18

**Authors:** Natthaporn Kaewchoothong, Sorracha Rookkapan, Chayut Nuntadusit, Surapong Chatpun

**Affiliations:** 1 Department of Mechanical and Mechatronics Engineering, Faculty of Engineering, Prince of Songkla University, Hat Yai 90112, Songkhla, Thailand; natthaporn.k@psu.ac.th (N.K.); chayut.n@psu.ac.th (C.N.); 2 Department of Radiology, Faculty of Medicine, Prince of Songkla University, Hat Yai 90110, Songkhla, Thailand; sorracha.r@psu.ac.th; 3 Department of Biomedical Sciences and Biomedical Engineering, Faculty of Medicine, Prince of Songkla University, Hat Yai 90110, Songkhla, Thailand

**Keywords:** abdominal aortic aneurysm, computational fluid dynamic, personalized model, hemodynamic changes, rupture risk

## Abstract

Abdominal aortic aneurysm (AAA) is a dangerous disease and can cause sudden death if it ruptures. This study investigated blood flow behaviors and hemodynamic changes in three categories (small, medium and large diameters) of AAAs using computational fluid dynamics (CFD) based on patient geometry. Computed tomography images of patients with abdominal aortic aneurysms were used to construct a patient-specific AAA model. This study included one healthy subject and seven patients who had AAAs with a diameter larger than 3 cm. The results showed that the aortic aneurysms were highly turbulent in the diastolic phase, and there was an increase in turbulence as the aneurysm size increased. The time-averaged wall shear stress (TAWSS) in the artery was high at peak systole and decreased during diastole. The oscillating shear index (OSI) was higher at the middle and distal aortic aneurysm sac than in other areas. Low TAWSS and a high OSI in the aneurysm region may indicate a risk of wall rupture in AAA. This study suggests that CFD provides further insights by visualizing blood flow behaviors and quantitatively analyzing hemodynamic parameters.

## 1. Introduction

Cardiovascular diseases are a group of harmful non-communicable diseases (NCDs) that are increasing in prevalence. These diseases are related to lifestyle behavior and their symptoms tend to accumulate gradually and become more severe if not treated properly and in time. Aneurysm is a dangerous disease and can cause sudden death if it ruptures. There are three major types of aneurysms: abdominal aortic aneurysm, thoracic aortic aneurysm and cerebral aneurysm. When an abdominal aortic aneurysm (AAA) occurs, the vascular wall of the aorta becomes weakened or enlarged or balloons outward. AAA is a chronic inflammatory condition and mostly found in older males who have one or more risk factors, such as emphysema, high blood pressure, high cholesterol, smoking or family history. Normally, an abdominal aorta is 15 mm to 24 mm in diameter, with the size depending on gender, age and weight [1,2]. In general, AAA is diagnosed when the abdominal aorta is larger than 30 mm [3]. It is often asymptomatic and discovered incidentally during abdominal imaging. A systematic review by Song et al. reported that the global prevalence of AAA among people aged 30 to 79 years was 0.92% [4]. AAA accounted for 2.19 deaths/100,000 people worldwide in 2017 [5]. A meta-analysis found that each 5 mm increase in aortic diameter increases the growth rate by 0.6 mm per year, leading to a risk of rupture [3]. Ultrasound screening for AAA is recommended for people at high risk.

Currently, computational fluid dynamics (CFD) plays an important role in understanding cardiovascular diseases, including aneurysms. Various studies have applied CFD simulation to investigate the hemodynamics of flow in patients with brain aneurysm and AAA [6,7,8,9,10]. These studies determined wall shear stress derivatives, wall pressure, flow behaviors and rupture risk. Furthermore, CFD based on patient-specific models has been performed for aneurysm rupture prediction, as well as aneurysm formation and risk assessments [9,11,12,13]. Qiu et al. performed CFD to classify the blood flow patterns in AAA and found that the helical main flow channel with helical vortices was associated with an increased risk of aneurysm rupture [7]. Based on CFD analysis, the wall shear stress in the symptomatic AAA group was lower than that in the asymptomatic AAA group [9]. Furthermore, CFD analysis has been performed in cases of endovascular aneurysm repair (EVAR) using a series of ideal anatomical models based on computed thermography angiography (CTA) scan data to assess the morphological and hemodynamic changes in vessels after EVAR, providing a further reference for the option of clinical therapy [14]. Therefore, combining patient-specific models with CFD analysis is interesting and relevant for understanding pathological effects of and clinical interventions for AAA.

While patient-specific CFD analyses of AAA have been widely reported, our study emphasizes the use of CFD as an assisting tool for clinical investigation to characterize blood flow behavior in patient-specific models with varying severities of aneurysm. This study aimed to investigate blood flow behaviors and hemodynamic changes across three categories (small, medium and large diameters) of AAA during both the systolic and diastolic phases using CFD based on the patient’s geometry. The high-risk areas for wall rupture, which were associated with hemodynamic changes in the aneurysm sac, were determined in the patient-specific models.

## 2. Materials and Methods

### 2.1. Image Acquisition and Patient Specific AAA Model

**Figure 1:**
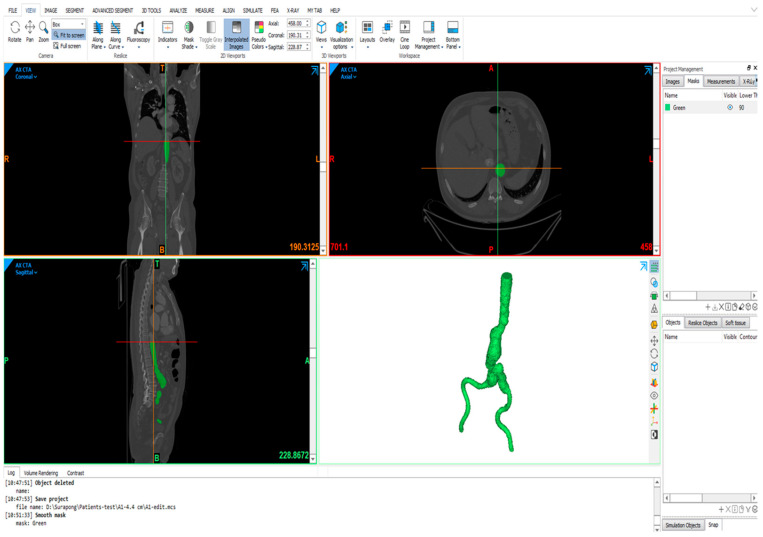
Three-dimensional vascular reconstruction of AAA using the Mimics v21.0 software.

**Figure 2:**
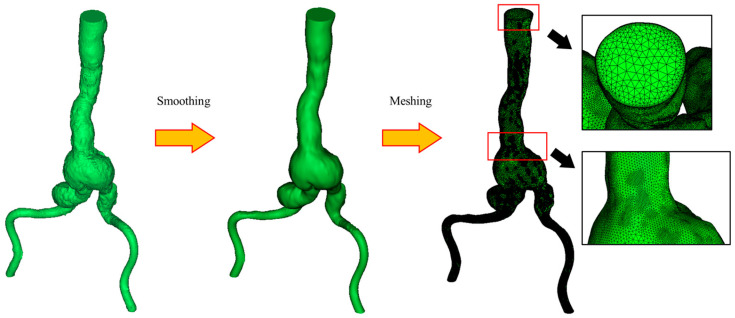
Smoothing and meshing for reconstructed AAA model.

The computed tomography (CT) images (DICOM file) of aorta from 7 male patients with AAA were obtained from the radiology department of Songklanagarind hospital. Three groups of models were determined based on the aneurysm sac diameter: (i) Group A with 3–5 cm in diameter, (ii) Group B with 5–7 cm in diameter, and (iii) Group C with a diameter above 7 cm. The three-dimensional (3D) reconstruction of AAA was performed using Mimics v21.0 (Materialise, Leuven, Belgium), as shown in Figure 1. Three techniques were applied to perform geometry reconstruction: thresholding, dynamic region growth and 3D computation. First, threshold masks were applied to select the region of interest based on Hounsfield units (gray values in CT images). The gray values are allocated to each pixel of CT images, and the densities of materials, including bones and vessels, are related to these values. Second, the dynamic region growth function was employed to split the masks into discrete portions for dealing with arteries, vessels and nerves. Third, the conversion from chosen areas to 3D AAA geometry was carried out using a 3D calculation tool. In addition, further smoothing and altering methods were used to tidy up the reconstructed geometry. The aortic-side branch arteries were eliminated to reduce the shape complexity and enable meshing and simulation. Finally, the 3D-smoothed geometry was obtained and exported in binary stereolithography format (.stl), as shown in Figure 2.

### 2.2. Governing Equations and Shear-Related Parameters

This study applied time-averaged governing equations contained mass and momentum conservation, which are represented as:

(1) $$\frac{\partial \rho }{\partial t}+\frac{\partial \rho u_{i}}{\partial x_{i}}=0$$

(2) $$\frac{\partial \rho u_{i}}{\partial t}+\frac{\partial \rho u_{i}u_{j}}{\partial x_{j}}=-\frac{\partial P}{\partial x_{i}}+\frac{\partial }{\partial x_{j}}\left[\left(2\nu S_{ij}-\rho \bar{u_{i}^{\prime }u_{j}^{\prime }}\right)\right]$$

where *u* is the velocity, ρ is the fluid density, *P* is the pressure, $S_{ij}$ is the strain tensor rate, *ν* is the dynamic viscosity, and $\bar{u_{i}^{\prime }u_{j}^{\prime }}$ is the Reynolds stress.

The SST k-ω turbulence model was used due to a blending function to achieve a smooth transition between the standard k-ω model near the wall and the k-ε model outside the boundary layer. Its turbulent kinetic energy (k) and specific dissipation rate (ω) transport equations are as follows:

(3) $$\frac{\partial \rho k}{\partial t}+\frac{\partial \rho u_{i}k}{\partial x_{j}}=P_{k}-\beta^{*}\rho \omega k+\frac{\partial }{\partial x_{j}}\left[\left(\mu +\sigma_{k}\mu_{t}\right)\cdot \frac{\partial k}{\partial x_{j}}\right]$$

(4) $$\frac{\partial \rho \omega }{\partial t}+\frac{\partial \rho u_{j}\omega }{\partial x_{j}}=\frac{\gamma \rho P_{k}}{\mu_{t}}-\beta \rho \omega^{2}+\frac{\partial }{\partial x_{j}}\left[\left(\mu +\sigma_{\omega }\mu_{t}\right)\cdot \frac{\partial \omega }{\partial x_{j}}\right]+2\left(1-F_{1}\right)\frac{\rho \sigma_{\omega 2}}{\omega }\frac{\partial k}{\partial x_{i}}\frac{\partial \omega }{\partial x_{i}}$$

where *F*_1_ is the blending function, which is defined as

(5) $$F_{1}=tanh\left[min{\left[max\left(\frac{2\sqrt{k}}{\beta^{*}\omega d},\frac{500\mu }{\rho d^{2}\omega }\right),\frac{4\rho \sigma_{\omega 2}}{CD_{k\omega }d^{2}}\right]}^{4}\right]$$

(6) $$CD_{k\omega }=max\left(2\rho \sigma_{\omega 2}\frac{1}{\omega }\frac{\partial k}{\partial x_{i}}\frac{\partial \omega }{\partial x_{i}},10^{-10}\right)$$

The model’s production terms are

(7) $$P_{k}=\tau_{ij}\frac{\partial u_{i}}{\partial x_{j}}=\left[\mu_{t}\left(2S_{ij}-\frac{2}{3}\frac{\partial u_{k}}{\partial x_{k}}\delta_{ij}\right)-\frac{2}{3}\rho k\delta_{ij}\right]\frac{\partial u_{i}}{\partial x_{j}}$$

The kinematic eddy viscosity is

(8) $$\mu_{t}=\frac{a_{1}k}{max\left(a_{1}\omega ,SF_{2}\right)}$$

The second blending function is defined as

(9) $$F_{2}=tanh\left[{\left[max\left(\frac{2\sqrt{k}}{\beta^{*}\omega y},\frac{500\nu }{y^{2}\omega }\right)\right]}^{2}\right]$$

The time-averaged wall shear (TAWSS) and oscillating shear index (OSI) are important parameters to predict disturbed flow conditions and plaque formation. The TAWSS determines the total shear stress that is exerted on the wall throughout a cardiac cycle and is computed as shown in Equation (10).

(10) $$TAWSS=\frac{1}{T}\int_{0}^{T}\left|WSS\;dt\right|$$

The OSI gives the zones where the WSS presents directional changes over the cardiac cycle, yielding 0 for uniaxial flows and 0.5 for no preferential direction. The equation for determining the OSI is Equation (11).

(11) $$OSI=0.5\times \left(1-\frac{\left|\int_{0}^{T}WSS\;dt\right|}{\int_{0}^{T}\left|WSS\right|dt}\right)$$

### 2.3. Numerical Simulation

The governing equations were solved, and the computations were performed using ANSYS Fluent 2020 R2 (ANSYS, Canonsburg, PA, USA). To solve the continuity and momentum equations based on a second-order upwind approach for spatial discretization, the pressure–velocity coupling was established as a SIMPLE (semi-implicit method for pressure-linked equation). The residual velocity and continuity convergence values were set at 1 × 10^−5^. The answers were calculated in 282-step cycles with 0.01 s timesteps.

### 2.4. Mesh Dependency Test

**Figure 3:**
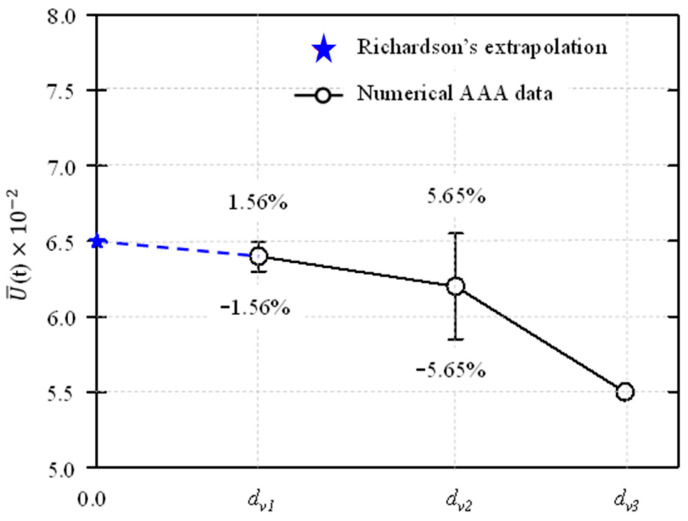
Mesh refinements compared with those obtained from Richardson extrapolation in the case of patient specific AAA model.

The meshes for the patient-specific AAA model were created using ICEM CFD 2020 R2 (ANSYS, Canonsburg, PA, USA). We utilized the previously authorized mesh convergence index to compute the computational simulation error [15,16]. Richardson extrapolation introduces computational error, which is captured by the grid convergence index. Three different mesh densities were evaluated to confirm mesh independence. Figure 2 shows the mesh refinement; the tetrahedral four-node elements are finer along the aortic wall. Mesh convergence was examined to assess the invariance of the numerical simulation results in relation to the mesh. The computations were carried out in parallel on a desktop computer powered by a 3.40 GHz quad-core CPU and 16.0 GB of RAM. Figure 3 shows the change in time-averaged magnitude velocity produced via Richardson extrapolation and different mesh refinement levels for the patient-specific AAA model.

Table 1 shows the mesh convergence findings. Because the volumes across simulations were comparable, the grid convergence index (GCI) was subjected to a mesh refinement ratio, mr_i_, that was equal to dv_i_/dv_i+1_. The CFD results are inside the asymptotic convergence range if the index ratio, GCI_3,2_/(rnGCI_2,1_), converges close to one. A rough ratio of 1.032 was found, showing mesh convergence; consequently, a finer mesh was not necessary.

### 2.5. Boundary Conditions

**Figure 4:**
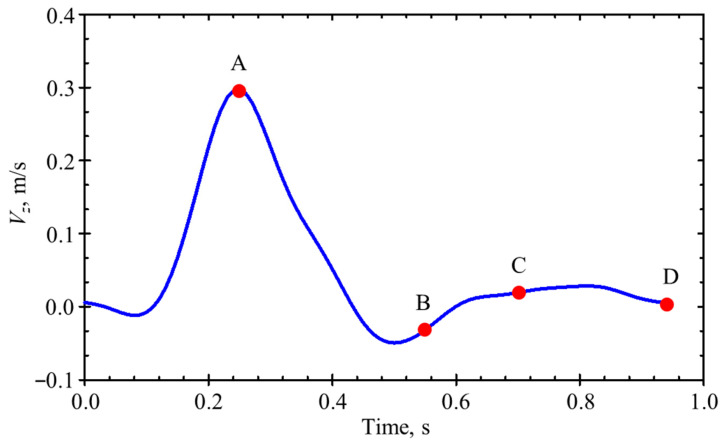
Initial velocity waveform in one cardiac cycle at the inlet of the AAA model. A: peak systole; B: early diastole; C: mid-diastole; D: late diastole.

Blood was considered a Newtonian fluid with a density of 1060 kg/m^3^ and a constant dynamic viscosity of 0.0035 Pa·s [17,18]. Because non-Newtonian effects are commonly found in small channels, the Newtonian fluid assumption is plausible in bigger blood vessels, such as aorta [18,19]. Furthermore, because the blood flow within arteries is pulsatile [20], a user-defined function for a time-dependent velocity waveform with a velocity range between −0.05 and 0.3 m/s was assigned at the inlet of the model during a cardiac cycle, as illustrated in Figure 4. The entrance velocity waveform defined in accordance with Rissland et al. [21]. At the common iliac arteries, the outlet boundary at zero-gauge pressure was employed [16,22]. The non-slip condition was applied on the artery wall.

## 3. Results

### 3.1. Reconstructed AAA Models

**Figure 5:**
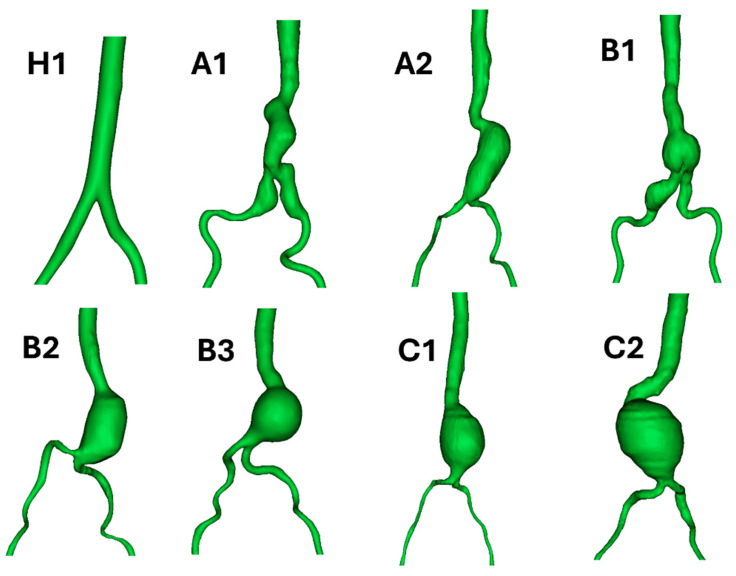
Reconstructed geometries of a healthy subject and AAA patients. Seven patient-specific models are categorized into three groups based on their abdominal aneurysm sac diameter: Group A, with 3–5 cm in diameter; Group B, with 5–7 cm in diameter; and Group C, with a diameter above 7 cm.

Seven patient-specific AAA models were grouped based on the aneurysm sac diameter and analyzed using CFD to investigate blood flow behaviors and hemodynamic changes. The diameter of the aneurysm sac and aneurysm characteristics of each reconstructed patient-specific model are presented in Table 2. All patients had fusiform AAAs with varied shapes. Patients A1 and B2 had not only abdominal aortic aneurysm but also Type I-iliac artery aneurysm, as shown in Figure 5.

### 3.2. Blood Flow Behaviors

**Figure 6:**
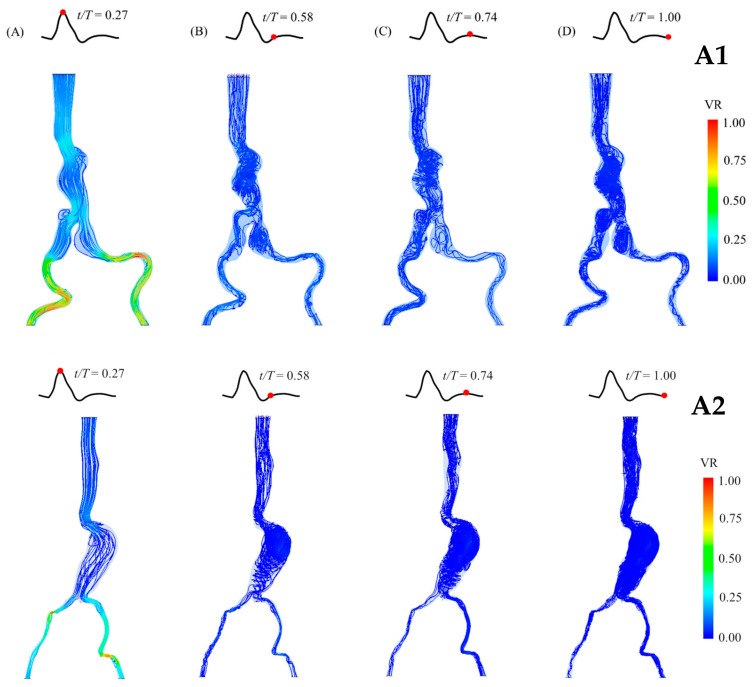
Blood flow behaviors in the patient-specific AAA model of Group A (aneurysm sac diameter of 3–5 cm) during a cardiac cycle (at different time points; (**A**) peak systole; (**B**) early diastole; (**C**) mid-diastole; (**D**) late diastole), showing the velocity streamlines and velocity ratio (VR). The model is presented in the front view. (**Top**): Patient A1, with 4.4 cm aneurysm sac diameter. (**Bottom**): Patient A2, with 4.6 cm aneurysm sac diameter.

**Figure 7:**
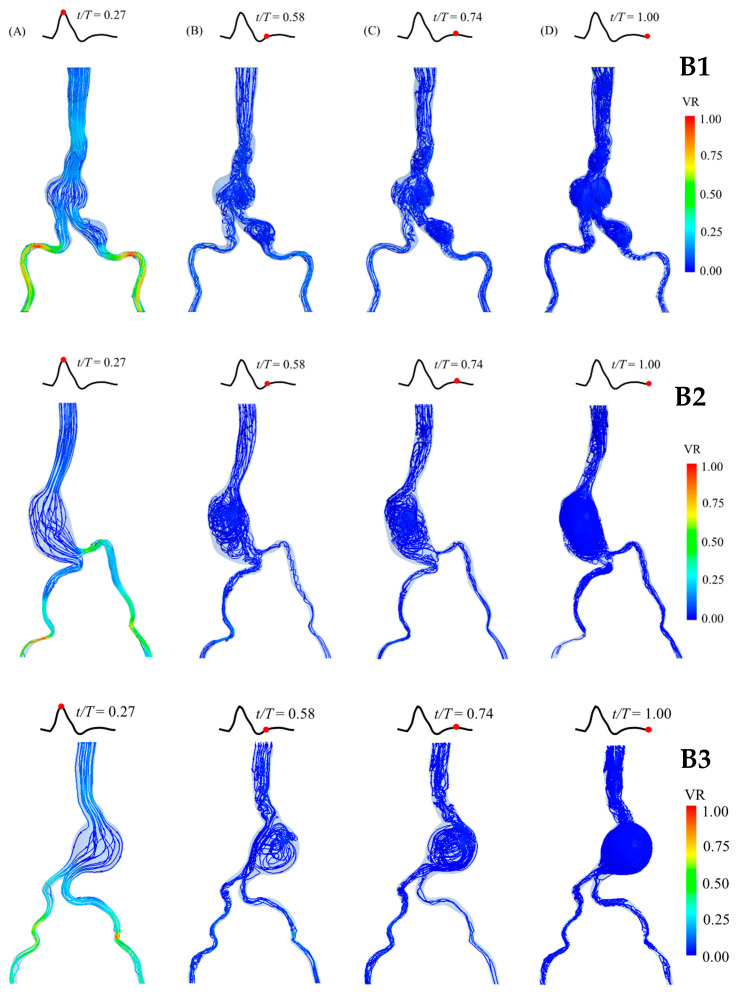
Blood flow behaviors in the patient-specific AAA model of Group B (aneurysm sac diameter of 5–7 cm) during a cardiac cycle (at different time points; (**A**) peak systole; (**B**) early diastole; (**C**) mid-diastole; (**D**) late diastole), showing the velocity streamlines and velocity ratio (VR). The model is presented in the front view. (**Top**): Patient B1, with 5.1 cm aneurysm sac diameter. (**Middle**): Patient B2, with 5.2 cm aneurysm sac diameter. (**Bottom**): Patient B3, with 6.5 cm aneurysm sac diameter.

**Figure 8:**
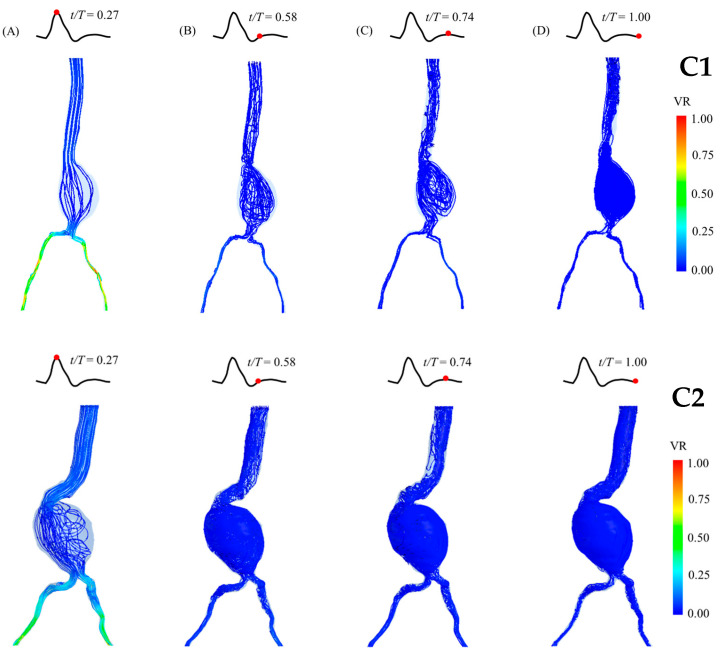
Blood flow behaviors in the patient-specific AAA model of Group C (aneurysm sac diameter larger than 7 cm) during a cardiac cycle (at different time points; (**A**) peak systole; (**B**) early diastole; (**C**) mid-diastole; (**D**) late diastole), showing the velocity streamlines and velocity ratio (VR). The model is presented in the front view. (**Top**): Patient C1, with 7.1 cm aneurysm sac diameter. (**Bottom**): Patient C2, with 8.4 cm aneurysm sac diameter.

**Figure 9:**
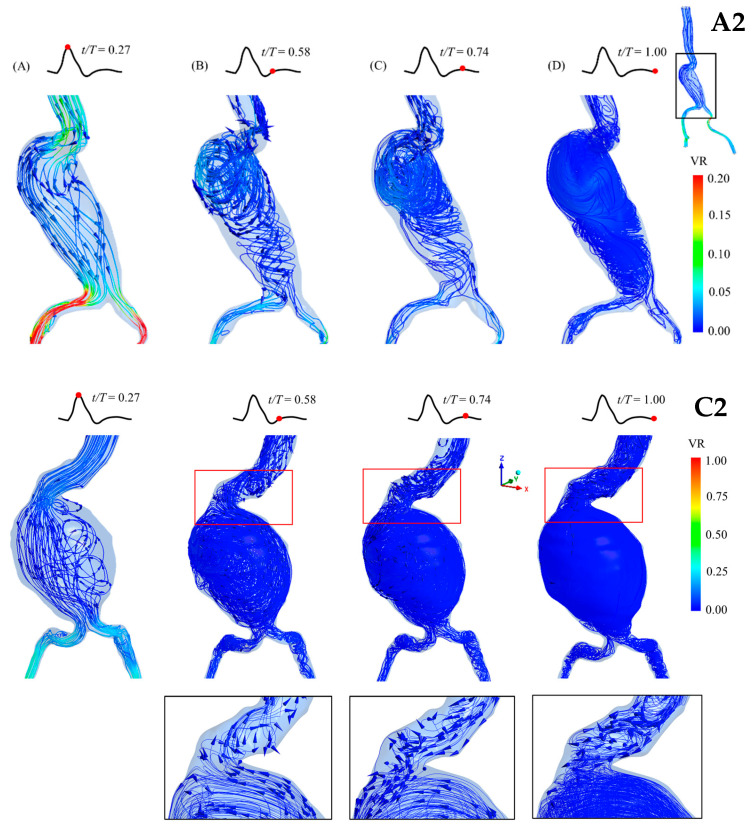
Magnification of flow patterns in the aneurysm sac of patient A2 with an aneurysm diameter of 4.6 cm (**Top**) and patient C2, with an aneurysm sac diameter of 8.4 cm (**Bottom**) at different time points; (**A**) peak systole; (**B**) early diastole; (**C**) mid-diastole; (**D**) late diastole.

The blood flow behaviors and velocity streamlines in the aneurysm sacs at four different time points of a cardiac cycle are presented for each group in Figure 6, Figure 7 and Figure 8. At the peak systole, the outlet at the iliac arteries has the highest velocity (velocity ratio (VR) = 1). Moreover, the streamline is not mixed up compared with the streamline in diastole. It can be clearly seen that the blood flow becomes turbulent and mixed up in the diastolic phase, especially at the end of diastole. It is also clearly shown that recirculation occurs in both abdominal and iliac aneurysms, as shown in the A1 and B1 models. Regarding the blood flow in the C2 model, which is the case with the largest diameter, a recirculation and mixing pattern can be observed in the aneurysm sac from the peak systole, which becomes more chaotic during diastole (Figure 9). At the neck of the aneurysm, the streamlines appear to retrograde and form vortices, indicating flow recirculation, especially during the mid-diastolic phase.

**Figure 10:**
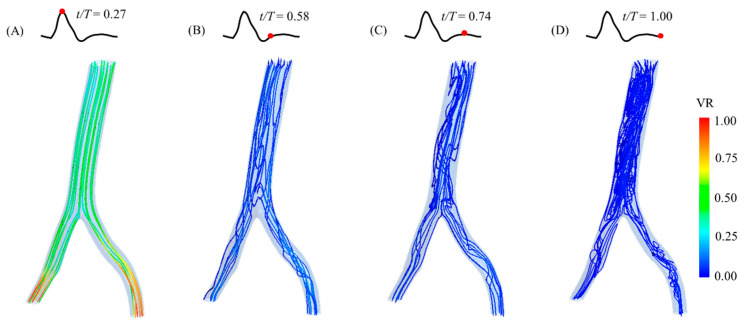
Blood flow behaviors in the healthy volunteer-specific model during a cardiac cycle (at different time points; (**A**) peak systole; (**B**) early diastole; (**C**) mid-diastole; (**D**) late diastole), showing the velocity streamlines and velocity ratio (VR). The model is presented in the front view.

In the healthy volunteer’s model, the velocity streamlines are fairly straight and parallel at the peak systole, as shown in Figure 10. The highest velocity ratios are observed at the iliac arteries. During the diastolic phase, even mixing and recirculating patterns are observed, and the velocity streamlines are relatively organized at the early diastole compared with the patterns observed in the AAA cases.

### 3.3. Hemodynamic Changes

**Figure 11:**
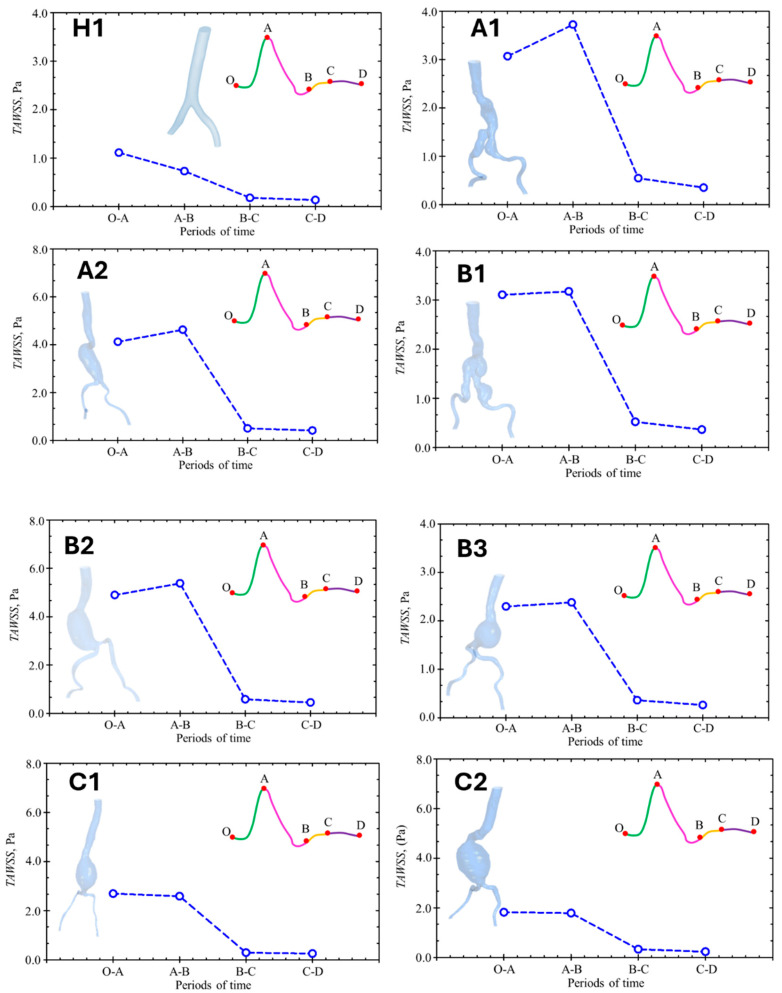
Time-averaged wall shear stress (TAWSS) in each model during a cardiac cycle.

In this study, the time-averaged shear stress (TAWSS) and oscillating shear index (OSI) were determined. Figure 11 shows the TAWSS of each model at each specific time points during a cardiac cycle. From early to peak systole (O-A period), the TAWSS reaches its highest value compared with other periods in a healthy subject, whereas in AAA patients (Groups A and B), the highest TAWSS values occurs from the peak systole to the early diastole (A-B period). The TAWSS in Group C, which has the largest aneurysm diameter, does not exhibit any significant difference between the early and peak systole or the peak systole and the early diastole. The TAWSS values in Groups A and B are 3–5 times higher than the value observed in the healthy volunteer, whereas the TAWSS in Group C is 2 times higher than that of the healthy volunteer.

**Figure 12:**
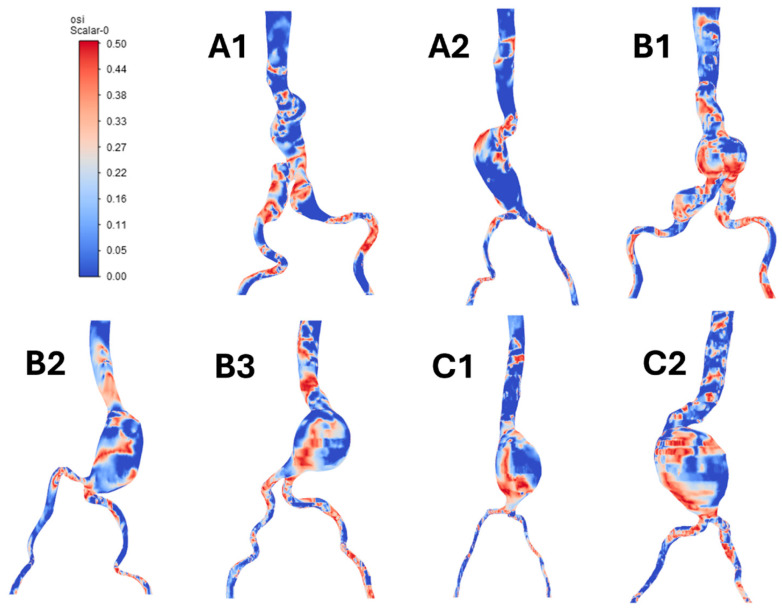
Oscillating shear index (OSI) in each model during a cardiac cycle.

Figure 12 shows the OSI of each model at specific time points during a cardiac cycle. It can be observed that high OSI values occur at the aneurysm areas, as well as at the bifurcation of iliac arteries, especially tortuosity parts. Furthermore, we can observe high-OSI regions (OSI > 0.38) at the middle and distal aneurysm near the bifurcation, rather than at the proximal part, for every patient group. Patients A1 and B1, who both had iliac aneurysms, exhibit similar OSI results at the bifurcation of iliac arteries. Furthermore, patients B1 and C2 exhibit areas with higher OSI values than the other patients, which may be attributable to their more complex geometry. The aneurysm with a higher angulated neck exhibits more high-OSI regions than those with a lower angulated neck, as seen in patient C1 versus patient C2.

## 4. Discussion

In this study, we applied CFD to investigate blood flow behaviors and hemodynamic changes in three different categories of AAA based on the patients’ aneurysm diameter. The current study extends the analysis of AAA to multiple real patient-specific cases with different sac diameters. This allows for a more comprehensive understanding of flow patterns and wall shear stress-related distribution in relation to aneurysm severity. The CFD results confirm that blood flow behavior during systole still has parallel streamlines, with minimal mixing between streamlines in the aneurysm sac. However, the mixing of streamlines increases with the diameter of the aneurysm, as shown in Group C. In contrast to diastole, recirculation and turbulent flow occur in both healthy and aneurysm cases. However, recirculation is more complex in the aneurysm sac than in the healthy case, even when the diameter of the aneurysm is small. The CFD simulation shows that the blood flow velocity is slowed down in the aneurysm sac and increases after passing through iliac arteries, which can cause flow disturbance and hemodynamic changes. The TAWSS is higher in the aneurysm cases than the healthy case, especially in the systolic phase. A large area with high OSI values occurs when the diameter of the aneurysm increases, a phenomenon that in particular leads to wall rupture.

The flow in the aneurysm sac decelerates and becomes instable because of the sudden expansion when the blood enters the aneurysm sac [23]. This phenomenon usually causes flow separation and vortex formation inside the aneurysm sac [24]. Qui et al. classified flow patterns in aortic aneurysm sacs into three types based on the structure of vortices [7]. They used CFD to simulate and validate these flow patterns in patients based on four-dimensional flow magnetic resonance imaging (4D MRI) scans. Our study reports flow patterns presenting as type I during systole, which is a non-helical main blood flow channel that impacts the aneurysm wall. In the diastolic phase, the blood flow pattern becomes a helical flow in the aneurysm sac, in contrast to the blood flow in healthy subjects. In our study, the geometry of the aneurysm sac and the neck angle are different in each case. This might lead to different blood flow patterns, even if the aneurysm diameter is categorized as being in the same group. Therefore, the geometric characteristics of a saccular aneurysm and the neck angle play important roles in flow patterns [25,26,27]. Furthermore, the presence of an iliac aneurysm sac affects the maximum velocity. When we compared the maximum velocity among the three groups, we found that the geometries with an iliac aneurysm sac demonstrated lower maximum velocities than those without one (case A1 (V_max_ = 2.312 m/s) vs. case A2 (V_max_ = 5.426 m/s) and case B1 (V_max_ = 2.972 m/s) vs. case B2 (V_max_ = 5.387 m/s)). Recirculation occurring in the aneurysm creates stagnant blood flow and leads to accumulation of platelets and clotting factors. This is the role of the vortical structure in intraluminal thrombus accumulation [28,29].

Wall shear stress (WSS) is the tangential frictional force that acts on the internal surface of blood vessels. Low-WSS areas are subjected to flow stagnation and secondary flow. It was reported that a low WSS correlated with an increased risk of aneurysm growth and rupture [30,31]. The results of several studies using CFD show that the WSS at the proximal and distal ends of an aneurysm sac exhibit high temporal variability and a heterogeneous spatial distribution [25,32,33]. Low-WSS regions correspond to areas with stagnation during the peak systolic phase and recirculating flow patterns during the end-diastolic phase. This flow pattern was consistently observed at rupture sites across all patient cases in the study by Moon et al. [34]. Although a low WSS can lead to thrombus formation, a high WSS can cause damage to endothelial cells. The TAWSS and OSI are WSS-related hemodynamic parameters that are used in monitoring AAA progression and rupture. The TAWSS is low in the aneurysm region, while the OSI is high in this region [34]. Our current study found that the TAWSS is high during the systolic phase and decreases during the diastolic phase, which is similar to the results of our previous study using CFD with idealized models [25]. The seven cases in our study presented high-OSI regions occurring at the middle and distal aneurysm near the bifurcation rather than at the proximal part, especially in Group C (with the largest aneurysm diameter). The larger the aneurysm diameter is, the larger the expansion is, and the higher the OSI is. This can result in areas with a high risk of aneurysm rupture [34,35,36,37]. Several recent studies have calculated the relative residence time (RRT) and endothelial cell activation potential (ECAP), which are key hemodynamic parameters, to be the same as the TAWSS and OSI [34,37,38]. A high RRT and elevated ECAP indicate potential high-risk areas for aneurysm rupture and thrombosis formation. Therefore, combining these hemodynamic parameters is beneficial for differentiating AAA characteristics and analyzing areas with high rupture risks.

Our results for the flow patterns, wall shear stress-related parameters and recirculation patterns are consistent with previously published patient-specific AAA studies [6,34,39,40]. This qualitative agreement supports the validity of our numerical simulations, including flow patterns and areas of high OSI values. This study can enhance our understanding of both qualitative and quantitative information concerning blood flow patterns in AAA during the deceleration phase and be used as a predictive tool for clinical evaluation. CFD assists clinical evaluation to explore fundamental hemodynamic changes and visualize recirculation areas that clinical and imaging methods are unable to emphasize. In clinical applications, CFD can be applied to assess rupture risk, ILT and plaque formations, endothelial dysfunction and wall remodeling. However, patient-specific geometry and boundary conditions, arterial thickness variation and heterogeneous material properties of the arterial wall should be considered in a comprehensive computational analysis. In addition, establishing the correlation between CFD results and clinical evaluation is essential to enhance model validity and promote real-world clinical translation.

This study has several limitations: First, it included limited cases of AAA patients in its CFD simulation, which might not enable general conclusions across the three categories of AAA. Increasing the number of AAA cases can give further insights into the blood flow behavior in the aneurysm sac and the risk of rupture. Second, the boundary conditions at the inlet and outlet were defined using generalized flow profiles rather than patient-specific measurements due to data availability. This could affect the accuracy of our simulation. To achieve more accurate results, future studies should input patient-specific flow data, which can be obtained from advanced imaging modalities such as 4D flow MRI or duplex ultrasound. Furthermore, using clinical image data such as Doppler ultrasound or phase-contrast MRI to validate the model’s results would be beneficial for confirming its predictive accuracy. Our models also assumed rigid vessel walls and blood as a non-Newtonian fluid, which does not represent the real properties of vessel walls and blood. A fluid–structure interaction model with Newtonian fluid properties should be applied to obtain a more realistic simulation of the blood flow in the compliant blood vessel. Furthermore, in this study, some hemodynamic parameters, such as wall shear stress gradient, pressure gradient, RRT and ECAP, were not determined. Calculating these parameters in future work would provide more comprehensive data and allow for better correlation with pathological conditions of AAA.

## 5. Conclusions

This study applied CFD combined with patient-specific models to investigate blood flow behaviors and hemodynamic changes in three categories of abdominal aortic aneurysms: small, medium and large diameter. From systole to diastole, the blood flow pattern becomes more turbulent and recirculatory. Increasing aneurysm diameters potentially affect the blood flow velocity, wall shear stress and other hemodynamic-related parameters, such as the TAWSS and OSI. Low TAWSS and high OSI values in the aneurysm regions potentially indicate a risk of wall rupture in AAA. This study suggests that CFD provides further insights by visualizing blood flow behaviors and quantitatively analyzing hemodynamic parameters.

## Figures and Tables

**Table 1:** Mesh convergence results from computational simulations of the patient-specific AAA models.

Mesh Type (i)	*d_vi_* (10^−3^)	*mr_i_*	$\bar{U}\left(t\right)$ m/s	GCI_i+1_
Fine (1)	2260	~1.47	0.064	1.56%
Intermediate (2)	1700	~1.47	0.062	5.65%
Coarse (3)	1050	-	0.055	-

**Table 2:** The diameter of the aneurysm sac and aneurysm characteristics of each reconstructed patient-specific AAA model.

Patient No.	Age (Year)	Gender	AAA sac Diameter (cm)	Aneurysm Characteristics
H1	52	M	-	no aneurysm
A1	67	M	4.4	at abdominal and iliac/with angulated neck
A2	66	M	4.6	at abdominal/with angulated neck
B1	80	F	5.1	at abdominal and iliac/with angulated neck
B2	69	M	5.2	at abdominal/with angulated neck
B3	87	F	6.5	at abdominal/with angulated neck
C1	65	M	7.1	at abdominal/without angulated neck
C2	82	M	8.4	at abdominal/with angulated neck

## Data Availability

The original contributions presented in this study are included in the article. Further inquiries can be directed to the corresponding author.
